# Jumonji domain-containing protein RIOX2 is overexpressed and associated with worse survival outcomes in prostate cancers

**DOI:** 10.3389/fonc.2023.1087082

**Published:** 2023-01-27

**Authors:** Chenchen He, Wang Liu, Jiahao Sun, Da Zhang, Benyi Li

**Affiliations:** ^1^ Department of Radiation Oncology, The First Affiliated Hospital, Xi’an Jiaotong University, Xi’an, China; ^2^ Department of Urology, The University of Kansas Medical Center, Kansas City, KS, United States; ^3^ Department of Pathology & Laboratory Medicine, The University of Kansas Medical Center, Kansas City, KS, United States

**Keywords:** RIOX2, prostate cancer, patient survival, disease progression, Myc protein

## Abstract

**Background:**

Histone demethylase RIOX2 was cloned as a c-Myc downstream gene involved in cell proliferation and has been implicated as an oncogenic factor in multiple tumor types. Its expression profiles and correlation with disease progression in prostate cancers are unknown.

**Methods:**

Transcriptomic profiles of Jumanji domain-containing protein genes were assessed using multiple public expression datasets generated from RNA-seq and cDNA microarray assays. RIOX2 protein expression was assessed using an immunohistochemistry approach on a tissue section array from benign and malignant prostate tissues. Gene expression profiles were analyzed using the bioinformatics software R package. Western blot assay examined androgen stimulation on RIOX2 protein expression in LNCaP cells.

**Results:**

Among 35 Jumanji domain-containing protein genes, 12 genes were significantly upregulated in prostate cancers compared to benign compartments. COX regression analysis identified that the ribosomal oxygenase 2 (RIOX2) gene was the only one significantly associated with disease-specific survival outcomes in prostate cancer patients. RIOX2 upregulation was confirmed at the protein levels using immunohistochemical assays on prostate cancer tissue sections. Meanwhile, RIOX2 upregulation was associated with clinicopathological features, including late-stage diseases, adverse Gleason scores, TP53 gene mutation, and disease-free status. In castration-resistant prostate cancers (CRPC), RIOX2 expression was positively correlated with AR signaling index but negatively correlated with the neuroendocrinal progression index. However, androgen treatment had no significant stimulatory effect on RIOX2 expression, indicating a parallel but not a causative effect of androgen signaling on RIOX2 gene expression. Further analysis discovered that RIOX2 expression was tightly correlated with its promoter hypomethylation and MYC gene expression, consistent with the notion that RIOX2 was a c-Myc target gene.

**Conclusion:**

The Jumanji domain-containing protein RIOX2 was significantly overexpressed in prostate cancer, possibly due to c-Myc upregulation. RIOX2 upregulation was identified as an independent prognostic factor for disease-specific survival.

## Introduction

The Jumanji domain-containing proteins are a family of histone modification enzymes involved in modulating gene expression towards cell differentiation, proliferation, and stress response (1, 2). This family of proteins contains multi-subgroups with various features of histone modification sites and domain structure properties (3, 4). Altered expression of these proteins has been reported in multiple human cancers (1), including prostate cancer (5–7).

Prostate cancer is a significant health issue in western countries, and it is the second leading cause of cancer death in American men, behind only lung cancer (8). About one man in 8 will be diagnosed with prostate cancer during his lifetime, and about one man in 41 will die of prostate cancer in the US, according to the ACS website description (www.cancer.org). Although the 5-year survival rate for localized diseases is about 100%, this rate drops to 30% for metastatic illnesses (9). Metastatic prostate cancers are initially treated with androgen deprivation therapy (ADT) because androgen and its cognate androgen receptor (AR) are critical for prostate cancer growth and progression (10). Castration by surgery or medical treatment reduces androgen levels, resulting in prostatic atrophy and prostate cancer regression. Unfortunately, metastatic prostate cancers often relapse and progress to a stage termed castration-resistant prostate cancers (CRPC) after 1.5-2 years of androgen deprivation therapy (11). Current CRPC treatments focus on suppressing AR activity with antagonists like Enzalutamide/Apalutamide or reducing androgen production from the adrenal gland and prostate cancer cells with Abiraterone (12). However, these treatments fail to yield a meaningful benefit in CRPC patients due to AR gene mutations or splice variations (i.e., AR-v7) (10), or cross-activation by other cellular signal pathways, resulting in AR re-activation (13). Therefore, novel diagnostic and therapeutic strategies are urgently needed to manage prostate cancers efficiently.

To develop novel strategies for prostate cancer management, we conducted a series of transcriptomic analyses using public RNA-seq datasets to explore new prognostic and therapeutic biomarkers (14, 15). Since multiple Jumonji domain-containing proteins were reported to modulate AR activity in prostate cancers, we analyze the expression profiles of 35 Jumanji domain-containing genes and their association with clinicopathological features in this study. Among these genes, twelve showed a significant alteration (nine upregulated and three downregulated) at the mRNA level in primary prostate cancer tissues compared to benign compartments. Most significantly, the c-Myc target gene RIOX2 was upregulated and tightly associated with disease progression and patient survival outcomes, representing a novel prognostic factor in prostate cancer.

## Materials and methods

### Gene expression profile analysis

Gene expression profiles of 35 Jumanji domain-containing protein genes at the mRNA level were analyzed using the RNA-seq dataset obtained from the Cancer Genome Atlas Prostate Adenocarcinoma project (TCGA-PRAD). There were 499 patients with primary prostate cancers in this cohort, of which case-matched benign specimens from the prostate glands were obtained from 52 cases. The RNA-seq data in Fragments Per Kilobase per Million (FKPM) format were downloaded from the TCGA portal (https://portal.gdc.cancer.gov) and were then converted to Transcripts Per Million (TPM) reads format. Finally, the data were presented as log_2_ [TPM + 1] for analysis. All analyses were conducted using the R-language package (version 3.6.3) on the bioinformatics platform (www.xiantao.love). RIOX2 gene expression profile was also assessed using the MSKCC dataset from 126 prostate cancers (16) and SU2C/PCF dataset from 429 patients (17) on the cBioportal platform (www.cbioportal.org).

### Kaplan-Meier survival assessments

The association of RIOX2 gene expression with patient survival outcomes was assessed using the Kaplan-Meier curve approach. Patients were divided into groups with high or low RIOX2 gene expression using the minimum *p*-value strategy (18). The Log-rank test was utilized to determine the significance of the hazard ratio (HR).

### Immunohistochemistry analysis

RIOX2 protein expression was assessed using an immunohistochemistry (IHC) approach on tissue microarray slides commercially obtained from Novus Biologicals, LLC (Avenue, CO). The tissue microarray contained 49 tissue sections derived from prostate cancer patients, of which there were nine case-matched pairs of normal and tumor sections. There were two cases of a Gleason score of 6 or 10, 15 cases of a Gleason score of 7 or 9, and 6 cases of a Gleason score of 8. The anti-RIOX2 polyclonal antibody was obtained from Sigma-Aldrich (St Louis, MO) and validated by the Human Protein Atlas project (HPA008080). The VECTASTAIN Elite kit (catalog #PK8200) from Vector Labs (Burlingame, CA) was used to visualize the immunosignal. A semi-quantitative approach was utilized to analyze the positive immunosignals, as described in our previous publication (19).

### Androgen modulation of RIOX2 expression in prostate cancer cells

RIOX2 expression in LNCaP cells after androgen stimulation or deprivation was analyzed using the NCBI GEO datasets (GDS3358, GDS2728, and GDS3111). For androgen deprivation, LNCaP cells were cultured in 10% charcoal-stripped fetal bovine serum (cFBS) (testosterone < 0.03 ng/ml) for up to 48 weeks. RIOX2 gene expression was assessed using the Affymetrix Human Genome U133 Plus 2.0 arrays (Affymetrix, Santa Clara, CA), as described in a previous publication (20). For androgen stimulation, LNCaP cells were cultured in 8% cFBS for three days and then treated with dihydrotestosterone (DHT, 10 nM) with or without the AR antagonist Casodex (10 nM). RIOX2 gene expression was examined using the Affymetrix Human Genome U133AB platform (21, 22).

To evaluate the androgen effect on RIOX2 expression at the protein level, LNCaP cells were treated with synthetic androgen R1881 in 2% cFBS with or without anti-AR drugs Enzalutamide and Abiraterone. R1881 was obtained from ICN (Aurora, OH) and dissolved in absolute Ethanol. Enzalutamide and Abiraterone were purchased from Cayman Chemicals (Ann Arbor, MI) and dissolved in dimethyl sulfoxide. Total cellular proteins were subjected to SDS-PAGE electrophoresis, followed by anti-RIOX2 immunoblotting assay (HPA008080, Sigma-Aldrich Co., St Louis, MO), as described in our publication (14).

### Statistical analysis

RNA-seq data were presented as a Log_2_ [TPM + 1]) value. Data in each group were also shown with the MEAN plus/minus the SEM (standard error of the mean). ANOVA test was used for multiple group comparisons, and a Student *t*-test was conducted to determine the significance of the differences between the two groups. Data visualization was performed using the R packages (version 3.6.3) and GraphPad software (version 9.1.0).

## Results

### Multiple Jumonji domain-containing proteins were upregulated in prostate cancers

**Figure 1:**
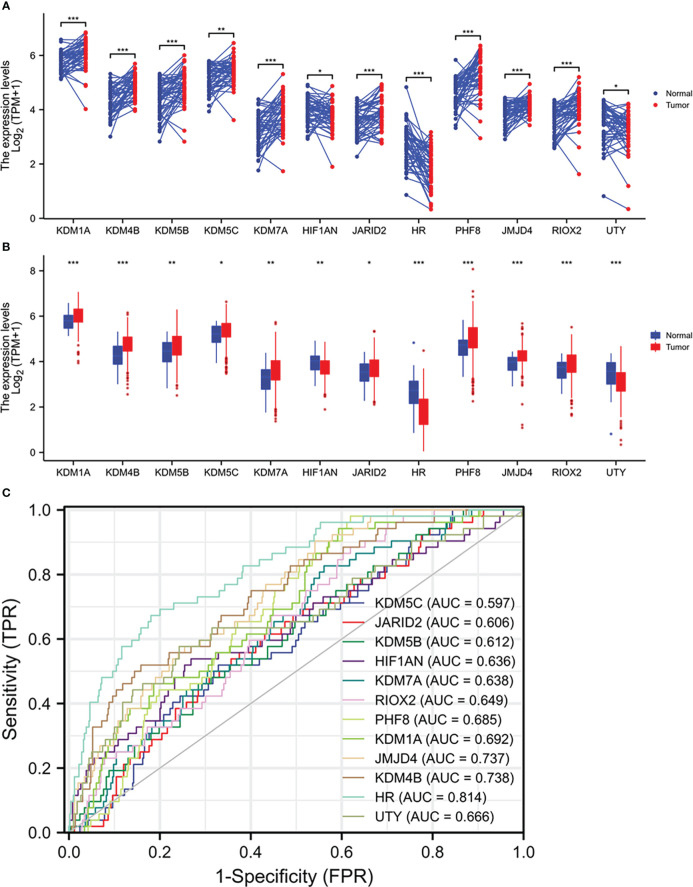
Multiple Jumanji domain-containing protein genes are upregulated in prostate cancers. **(A, B)** Gene expression at the mRNA levels was compared between normal and tumor tissues using the TCGA-PRAD RNA-seq dataset with a case-matched pairwise approach (panel A, n = 52, paired *t*-test) or group cohort approach (panel B, normal n = 52; tumor = 499, Wilcoxon rank sum test). The asterisks denote different significance levels, *p < 0.05, **p < 0.01, ***p < 0.001). **(C)** ROC analysis was conducted for the altered genes distinguishing normal and tumor tissues.

We analyzed the expression profiles of 35 Jumanji domain-containing protein genes in prostate cancers, and the list of genes was derived from two review articles (1, 2). We conducted two types of comparisons, case-matched pair of 52 benign-malignant tissues and a group cohort (benign *vs.* malignant) using the TCGA-PRAD dataset. Our results showed that nine genes were significantly upregulated, and three genes were significantly downregulated in both types of comparisons (**Figures 1A, B**). In contrast, the other 23 genes showed no significant or inconsistent differences between the two comparisons (**Figures S1A-D**). ROC analysis revealed that except for KDM5C (AUC = 0.597), all other genes exhibited a potent AUC value (> 0.6) in distinguishing malignant tissue from the benign compartment (**Figure 1C**). Specifically, the gene HR, a lysine demethylase and nuclear receptor corepressor, showed an AUC value of 0.814, representing a potential diagnostic marker for prostate cancer.

### RIOX2 upregulation was an independent prognostic factor of disease-specific survival

We then analyzed these altered genes’ association with disease-specific survival outcomes in prostate cancer patients. With five clinicopathological factors, COX regression analysis revealed that only RIOX2 upregulation and patient serum PSA level was significantly associated with disease-specific survival in the univariate test (**Table S1**). After eliminating other genes, RIOX2 upregulation showed a significant association with disease-specific survival in univariate and multivariate tests (**Table 1**). These data suggest that RIOX2 is an independent prognostic factor in prostate cancer, like serum PSA level.

**Table 1:** COX regression analysis of RIOX2 expression with disease-specific survival.

Characteristics Total (n)		Univariate analysis HR (95% CI)	P value	Multivariate analysis HR (95% CI)	P value
**T stage**	490				
T2	189	Reference			
T3&T4	301	519428284.120 (0.000-Inf)	0.999		
**N stage**	424				
NO	346	Reference			
N1	78	8.116 (0.736-89.560)	0.087	25.882 (0.984-680.484)	0.051
**PSA (ng/ml)**	440				
<4	413	Reference			
>=4	27	32.707 (5.137-208.243)	**<0.001**	27.624 (1.553-491.242)	**0.024**
**Gleason score**	497				
6&7	293	Reference			
8&9&10	204	892211776.881 (0.000-Inf)	0.999		
**Residual tumor**	466				
RO	315	Reference			
R1&R2	151	5.865 (0.609-56.523)	0.126		
**RIOX2**	497	10.409 (1.778-60.923)	**0.009**	42.361 (1.055-1700.569)	**0.047**

Bold font denotes a statistical significance.

**Figure 2:**
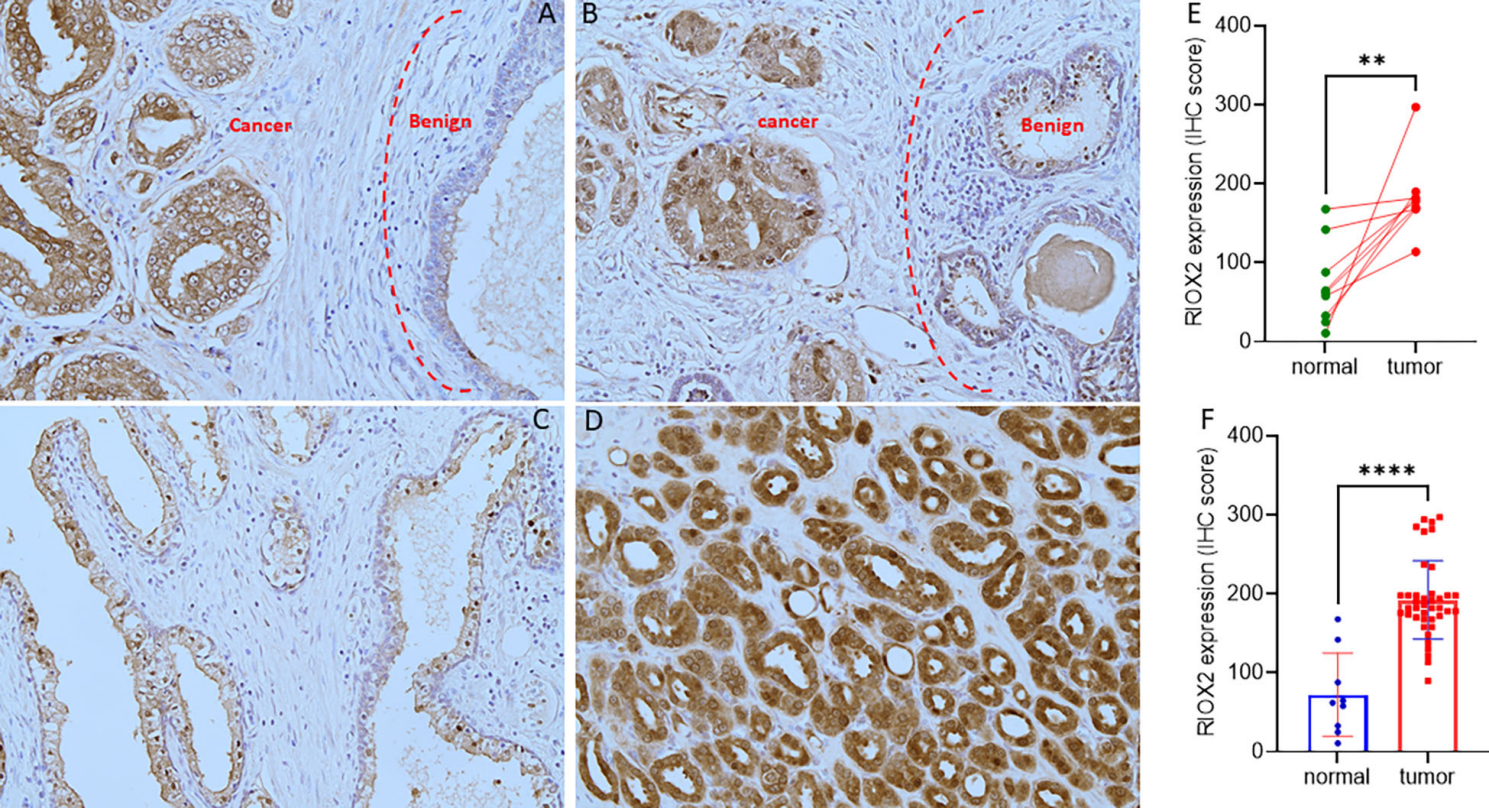
RIOX2 protein expression is increased in prostate cancer tissues. RIOX2 protein expression was examined in prostate cancer tissues with an IHC approach, as described in our recent publication (19). Representative microscopic images were shown from selected tissue sections with benign **(A–C)** and malignant compartments **(A, B, D)**. Magnification x 200. Semi-quantitative data (MEAN and SEM) of the immunosignals were shown for the pairwise **(E)** or group cohort comparison **(F)**. The asterisk indicates a significant difference (Student *t*-test, **p < 0.01, ****p < 0.0001).

To verify RIOX2 upregulation at the protein level, we conducted an immunohistochemical assay using a prostate cancer tissue array containing 40 cases. Of these tissue sections, there were nine case-matched benign-malignant tissue pairs. As shown in **Figure 2**, RIOX2 protein immunosignals were significantly increased in malignant tissues compared to their benign compartments. Semi-quantitative analysis of the immunosignals revealed a drastic difference in pairwise (**Figure 2E**) and group cohort comparisons (**Figure 2F**). These data indicated that RIOX2 protein expression was also upregulated in prostate cancers.

### RIOX2 expression was associated with cancer genetic alteration

**Figure 3:**
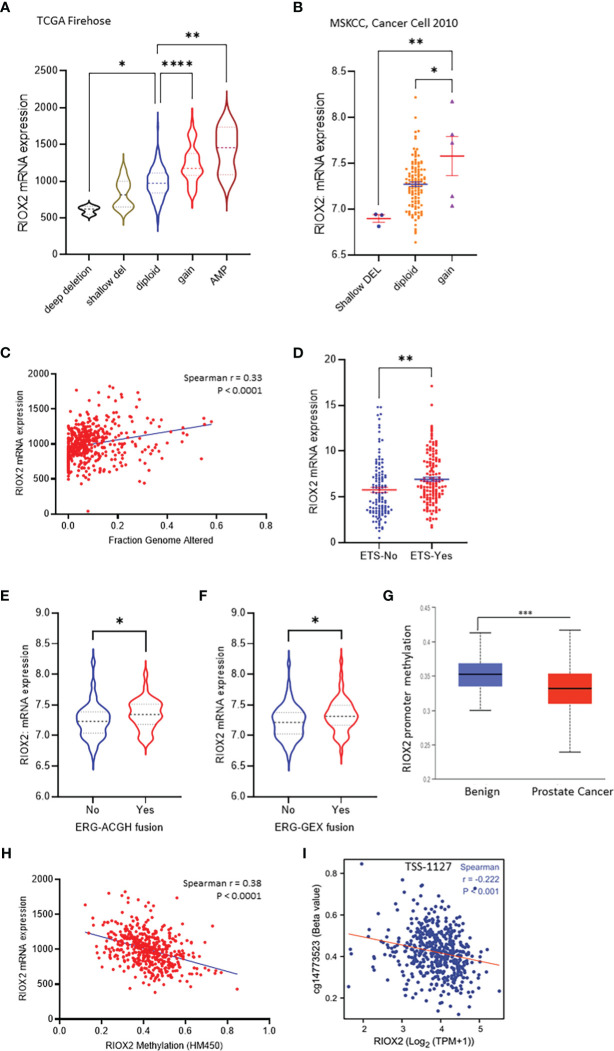
RIOX2 upregulation is associated with genetic alteration and promoter hypomethylation in prostate cancers. **(A)** RIOX2 mRNA expression was analyzed using the TCGA-PRAD RNA-seq dataset coupled with genetic copy number alterations. Case numbers in each group are as follows: deep deletion, 3; shallow deletion, 10; diploid, 430; gain, 45; amplification, 3. **(B)** RIOX2 mRNA expression was analyzed using the MSKCC RNA-seq dataset coupled with genetic copy number alterations. Each group’s case numbers are as follows: shallow deletion, 3; diploid, 118; gain, 5. **(C)** Spearman correlation analysis was performed using the TCGA-PRAD dataset between RIOX2 mRNA expression and Fraction Genomic Altered index. **(D–F)** RIOX2 mRNA expression levels in the TCGA-PRAD dataset were compared between groups with or without gene fusion features as indicated. The asterisk indicates a significant difference (Student *t*-test, *p < 0.05, **p < 0.01, ***p<0.001, ****p < 0.0001). **(G)** Promoter methylation levels of RIOX2 gene promoter were compared between benign and malignant prostate tissues using the UALCAN platform. **(H, I)** Spearman correlation analysis was conducted using the TCGA-PRAD RNA-seq and DNA methylation HM450 dataset.

Genomic alterations in cancer cells include mutations, copy number variations (CNV), and gene fusions. These alterations indicate tumor heterogeneity and homogeneity and are important factors associated with gene expression and cancer progression. We then examined the genetic alterations of the RIOX2 gene using the TCGA-PRAD RNA-seq dataset. Among 491 cases, only three (0.6%) showed genetic deep deletion or amplification. There were 10 cases with shallow deletion (2%) and 45 with genetic gain (9.16%). Two patients showed point mutations; one was A344V, and the other had two simultaneous point mutations, R197H & E447D. Consistently, RIOX2 mRNA levels were significantly increased along with the genetic gain and amplification (**Figure 3A**). Similar results were also observed from the MSKCC dataset (16); RIOX2 mRNA levels were significantly higher in prostate cancers with genetic gain (**Figure 3B**). In addition, RIOX2 mRNA levels were strongly correlated with the fraction value of genomic alterations in prostate cancers. Patients with ETS fusion, ERG-ACGH fusion, and ERG-GEX fusion all showed an increased level of RIOX2 mRNA (**Figures 3C–F**).

Since the low incidence (< 10%) of RIOX2 genetic gain/amplification was unlikely a driving cause of RIOX2 upregulation in prostate cancers, we turned to epigenetic alterations. We examined the promoter methylation levels within the RIOX2 gene locus. Our results showed that RIOX2 promoter methylation was significantly lower in cancer tissues compared to benign tissues (**Figure 3G**). A very strong reverse correlation was noticed between RIOX2 promoter methylation and mRNA levels in cancer tissues (**Figure 3H**). Further analysis revealed that the methylation intensity around the -1127bp region upstream of the transcription start site (TSS) was significantly correlated with RIOX2 mRNA levels (**Figure 3I**), indicating a potential mechanism of epigenetic modification.

### RIOX2 upregulation was associated with disease progression and cancer-specific survival

**Figure 4:**
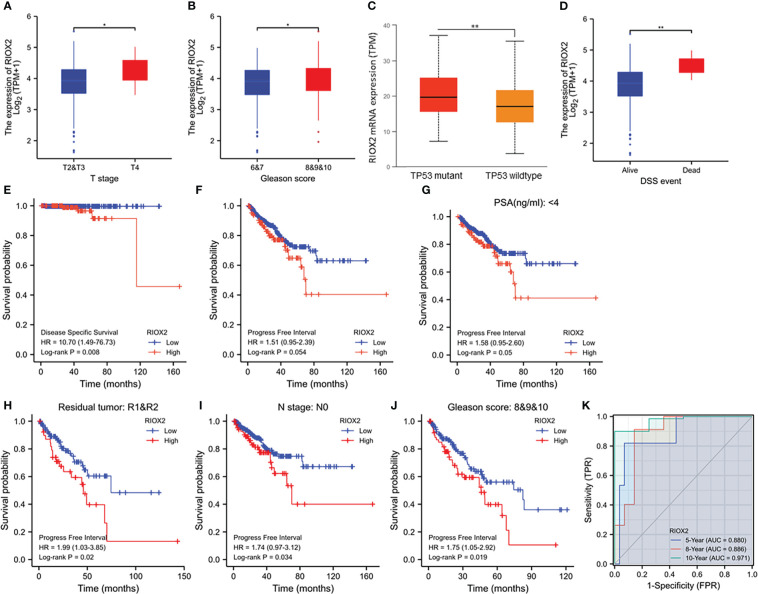
RIOX2 upregulation is associated with disease progression and patients’ survival outcomes in prostate cancers. **(A–D)** RIOX2 mRNA expression levels were compared in the TCGA-PRAD dataset between different groups stratified with tumor stage, Gleason scores, TP53 gene mutation status, and patient survival status as indicated. The asterisks indicate a significant difference (Wilcoxon rank sum test, *p < 0.05; **p < 0.01). Case numbers in each group are as follows: T2, 189; T3, 292; T4, 11; Gleason scores 6, 46; Gleason scores 7, 247; Gleason scores 8, 64; Gleason scores 9, 138; Gleason scores 10, 4; TP53 mutant, 38; TP53 wild-type, 295; DSS alive, 492, DSS deceased, 5. **(E–J)** Kaplan-Meier survival analysis was conducted by stratifying patients using the minimum *p*-value approach (18) based on RIOX2 expression levels and clinicopathological features. TMEM158 expression data were extracted from the TCGA-PRAD RNA-seq dataset and the **(K)** ROC analysis was conducted using the TCGA-PRAD dataset for the potential of RIOX2 mRNA expression on patient survival prognosis.

We analyzed the association of RIOX2 expression with patient clinicopathological features. Our results showed that higher levels of RIOX2 expression were seen in patients with late-stage (T4 *vs.* T2/3, **Figure 4A**), higher Gleason scores (8-10 *vs.* 6-7, **Figure 4B**), and tumors with TP53 mutation (**Figure 4C**). Deceased patients due to prostate cancer also showed a significantly higher level of RIOX2 mRNA levels compared to alive patients (**Figure 4D**). Kaplan-Myer curve analysis also showed that RIOX2 upregulation was significantly associated with disease-specific survival outcomes (**Figure 4E**). However, the association of RIOX2 expression with progression-free interval was at the borderline of significance (Log-rank p = 0.054, **Figure 4F**). However, after stratification of patients into subgroups, RIOX2 expression was significantly associated with the progression-free interval in patients with PSA level below 4 ng/ml (**Figure 4G**), with residue tumors after surgery (**Figure 4H**), without lymph node invasion (**Figure 4I**), or with higher Gleason scores (**Figure 4J**). ROC analysis indicated that RIOX2 expression is a robust prognostic factor (AUC = 0.88 - 0.971) of disease-specific survival for 5-10 years (**Figure 4K**).

### RIOX2 expression was positively associated with AR activity in CRPC patients

**Figure 5:**
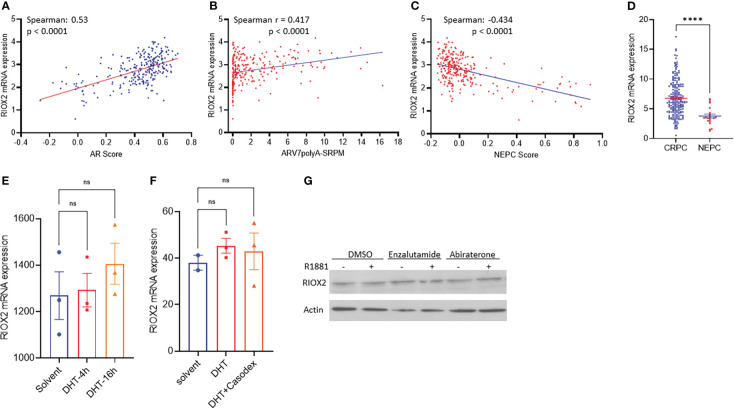
RIOX2 upregulation is associated with AR signaling activity in CRPC cancers. **(A–C)** Spearman correlation analysis was conducted between RIOX2 expression levels and AR signaling activity index, AR-V7 splicing, and NEPC index using the SU2C/PCF Dream Team RNA-seq dataset (17). **(D)** RIOX2 mRNA expression levels were compared between CRPC and NEPC tissues using the SU2C/PCF dataset. The asterisks indicate a significant difference (Student *t*-test, ****p < 0.0001). Case numbers: CRPC, 210; NEPC, 22. **(E, F)** RIOX2 mRNA expression data were extracted from NCBI GDS2782 (22) and GDS3111 (21). LNCaP cells were stimulated with dihydrotestosterone (DHT, 10 nM) with or without the AR antagonist Casodex (10 μM) in 8% cFBS-containing culture media. RIOX2 gene expression was examined using the Affymetrix Human Genome U133AB platform (21, 22). **(G)** LNCaP cells were treated with R1881 (1.0 nM) in 2% cFBS with or without Enzalutamide or Abiraterone (10 μM) overnight. RIOX2 protein expression was assessed in western blot assay (14). Actin blot served as a protein loading control. ns, none significance.

The clinical obstacle in managing metastatic prostate cancers is the castration-resistant progression after androgen deprivation therapy. Due to the vast implication of androgen receptor antagonists for castration-resistant prostate cancers (CRPC), neuroendocrinal progression of prostate cancer (NEPC) emerged as the worst stage of prostate cancer without means to cure (23, 24). We analyzed RIOX2 expression profiles in CRPC and NEPC specimens using the SU2C/PCF dataset (17). Our results showed that RIOX2 expression was significantly correlated with AR score (**Figure 5A**), an AR signal activity index (17). Meanwhile, RIOX2 expression was also strongly correlated with the expression levels of the AR-V7 splicing variant (**Figure 5B**), a potent indicator of treatment resistance in CRPC patients (25). In contrast, a robust reverse correlation was observed between RIOX2 expression and NEPC score (**Figure 5C**), an index of NEPC progression (17). Consistently, RIOX2 mRNA levels were significantly lower in NEPC tumors than in CRPC tumors (**Figure 5D**).

To determine if RIOX2 expression was directly regulated by the androgen/AR signal pathway, we analyzed RIOX2 expression in prostate cancer LNCaP cells after androgen stimulation. As shown in **Figures 5E–G**, androgen stimulation up to 16-24 h had no significantly enhancing effect on RIOX2 expression at the mRNA (**Figures 5E, F**) and protein levels (**Figure 5G**) in LNCaP cells. The AR antagonists, Casodex (Bicalutamide), Enzalutamide, and Abiraterone, did not significantly impact RIOX2 expression. Consistently, RIOX2 mRNA expression was observed in all prostate cancer cell lines with or without AR expression (**Supplemental Figure S2**). These data indicated that RIOX2 expression might not be a direct target gene of the AR signal pathway.

### RIOX2 expression was positively correlated with c-MYC expression

**Figure 6:**
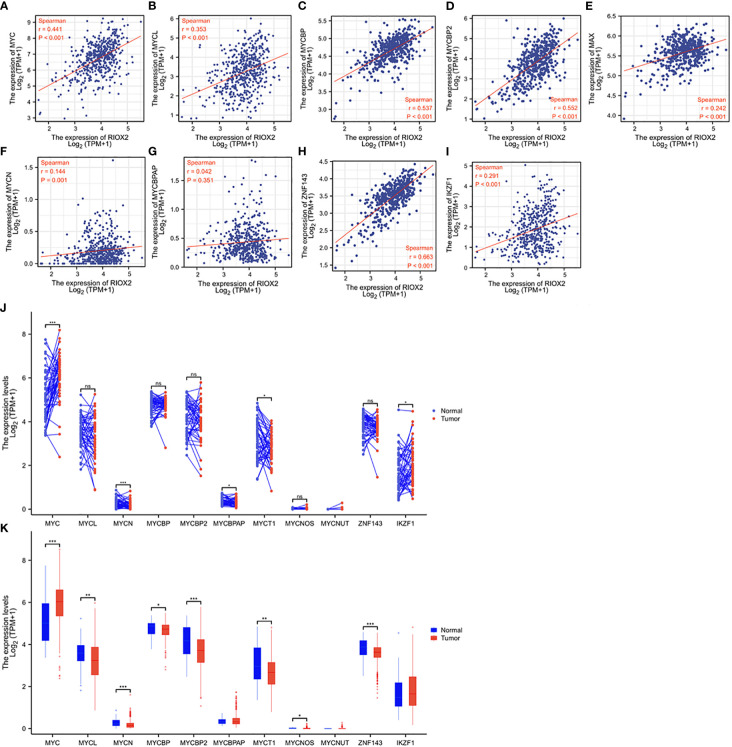
RIOX2 expression is correlated with MYC family genes in prostate cancer. **(A–I)** Spearman correlation analysis was conducted between RIOX2 and MYC family genes using the TCGA-PRAD RNA-seq dataset. Spearman r > +/- 0.3 was considered a strong correlation. **(J, K)** Gene expression levels of MYC family genes were extracted from the TCGA-PRAD RNA-seq dataset. Two types of comparison were conducted, case-matched pairwise **(J)** and group cohort (panel K) comparisons. The asterisk indicates a significant difference compared to the normal group (*p < 0.05, **p < 0.01, ***p < 0.001). ns, no significance. Case numbers: Normal tissues, 52; Tumors, 499.

RIOX2 gene was initially reported as a c-Myc downstream target involved in cell proliferation (26), and c-Myc is a proven oncogene in prostate cancer (27). Therefore, we examined the correlation of RIOX2 expression with the MYC family and its related genes. Consistent with a previous report (26), a strong positive correlation was found between RIOX2 expression and c-Myc (**Figure 6A**), L-Myc (**Figure 6B**), and Myc binding proteins (**Figures 6C, D**), while only a moderate correlation was noticed between RIOX2 expression and Myc associated factor-X (**Figure 6E**). In contrast, RIOX2 expression was not correlated with N-Myc (**Figure 6F**) and MYCBP-associated protein (**Figure 6G**). Interestingly, among these Myc family members, only c-Myc was significantly upregulated in prostate cancers compared to benign compartments (**Figures 6J, K**), suggesting that c-Myc is potentially a major regulator of RIOX2 expression in prostate cancer.

Recent reports showed that RIOX2 expression in human cancers was also modulated by zinc finger proteins ZNF143 (28) and IKZF1 (29) in liver cancers. We conducted a Spearman correlation analysis and found that RIOX2 expression was strongly correlated with ZNF143 expression (**Figure 6H**) while moderately with IKZF1 expression (**Figure 6I**) in prostate cancers. However, both ZNF143 and IKZF1 expression had no significant differences between malignant and benign tissues in prostate cancers (**Figures 6J, K**). These data suggest that ZNF143 or IKZF1 is unlikely an upstream modulator for RIOX2 upregulation in prostate cancer.

## Discussion

This study showed that 12 out of 35 Jumanji domain-containing protein genes were significantly altered at the mRNA level in primary prostate cancer tissues compared to the benign compartment. Among these 12 genes, only RIOX2 upregulation was tightly associated with tumor stage, Gleason score, TP53 gene mutation, and disease-specific survival in prostate cancer patients. Further analyses revealed that RIOX2 expression was correlated with its genetic gain/amplification and promoter hypomethylation, as well as fusion events of ETS and ERG genes. In CRPC patients, RIOX2 expression was positively correlated with the AR activity score and AR-V7 expression levels but negatively correlated with the NEPC score. Indeed, RIOX2 expression levels were significantly reduced in NEPC tumors compared to CRPC tumors. However, androgen stimulation had no significant enhancing effect on RIOX2 expression at both the mRNA and protein levels. These data suggest that RIOX2 was upregulated in prostate cancers and that its upregulation was associated with disease progression and cancer survival in parallel to AR activity levels.

Over the last two decades, genetic and epigenetic alterations have been identified as oncogenic drivers to modulate AR-dependent cistrome reprogramming during prostate cancer development, progression, and treatment resistance (30–32). Several epigenetic modifiers were identified as prostate cancer biomarkers, including histone demethylases (33). KDM1A was the first one reported to enhance AR signal activity (34), followed by KDM4C (35, 36) and KDM3A (37). Other histone demethylases, such as KDM3A (38, 39), KDM4A, KDM4D (40), KDM4B (41, 42), KDM7A (43), and JMJD6 (44), were also shown to enhance AR signal activity and to increase AR-v7 expression. In addition, several histone demethylases showed gene upregulation in prostate cancer tissues, including KDM1A (45), KDM3A and KDM4C (7), KDM4A (46), KDM4B (47), KDM5B (6), KDM5C (48), KDM6B (5, 49), KDM7A (43), and PHF8 (50), Among these upregulated genes, KDM4B expression was shown to correlate with patient overall survival outcomes, while KDM1A, KDM5C and KDM6B were shown to be associated with progression-free survival (45, 47–49).

In our analysis, twelve histone demethylases showed gene upregulation in prostate cancers, of which six (KDM1A, KDM4B, KDM5B, KDM5C, KDM7A, and PHF8) were in line with previous reports. Other six genes, including upregulated JARID2, JMJD4, and RIOX2, as well as downregulated HIF1AN, HR, and UTY were not reported in prostate cancers so far. COX regression analysis with multiple variates, including clinicopathological factors, revealed that only RIOX2 upregulation was significantly correlated with disease-specific survival in prostate cancer patients. ROC analysis indicated that RIOX2 upregulation is a strong prognostic predictor of ten-year disease-specific survival (AUC = 0.971). These results suggest that RIOX2 overexpression is a strong and independent prognostic factor for prostate cancer patients.

RIOX2 gene was initially discovered as a c-MYC downstream gene. Our analysis also confirmed this notion with convincing data that RIOX2 expression was tightly correlated with MYC family genes with a Spearman’s ρ higher than 0.4, including MYCBP2 (r = 0.552), MYCBP (r = 0.537), and c-MYC (r = 0.441). However, only c-MYC gene expression was significantly increased in prostate cancer tissues. These data indicated that c-Myc might be the significant factor in RIOX2 upregulation in prostate cancer. Further analysis discovered that RIOX2 expression was correlated with its promoter hypomethylation in prostate cancers, indicating an epigenetic mechanism involved in RIOX2 upregulation. In addition, RIOX2 expression was found to strongly correlate with ZNF143, which was previously shown to enhance RIOX2 gene expression in liver cancer cells (28). However, ZNF143 expression was not significantly altered in prostate cancer tissues, indicating it is unlikely a causative factor of RIOX2 upregulation in prostate cancer.

## Conclusion

We conducted a comprehensive transcriptomic analysis of 35 histone demethylases in prostate cancer and identified RIOX2 as a potent prognostic factor for disease-specific survival in prostate cancers. Further investigation is needed to understand its causative role in prostate cancer progression and its therapeutic value as a drug target.

## Data availability statement

The original contributions presented in the study are included in the article/**Supplementary Material**. Further inquiries can be directed to the corresponding author.

## Author contributions

BL designed the study. CH, JS, and BL analyzed the bioinformatics data. WL, DZ, and BL conducted the IHC. CH and BL drafted the manuscript. All authors contributed to the article and approved the submitted version.

## Supplementary material

Supplementary Figure 1 RIOX2 expression data in normal and malignant prostate tissues were extracted from the TCGA-PRAD dataset for two types of comparison, case-matched pairwise (panels A & C) and group cohort (panels B & D). Wilcoxon rank sum test, *p < 0.05; **p < 0.01; ***p < 0.001; ns, no significance.

Supplementary Figure 2 RIOX2 expression in normal and malignant prostate cell lines were extracted from the Cancer Cell Line Encyclopedia datasets (51, 52) downloaded from the cBioportal platform. Relative RIOX2 mRNA expression compared to the benign PRECLH cell line was shown after normalized with ACTB expression level.

Supplementary Table 1 COX aggression analysis of twelve Jumanji domain-containing protein genes with disease-specific survival outcomes.
