# Supplementary figures and images for: Jumonji domain-containing protein RIOX2 is overexpressed and associated with worse survival outcomes in prostate cancers
Source: Front Oncol. 2023 Jan 27;13:1087082. doi: 10.3389/fonc.2023.1087082 (PMC9911806; doi:10.3389/fonc.2023.1087082)

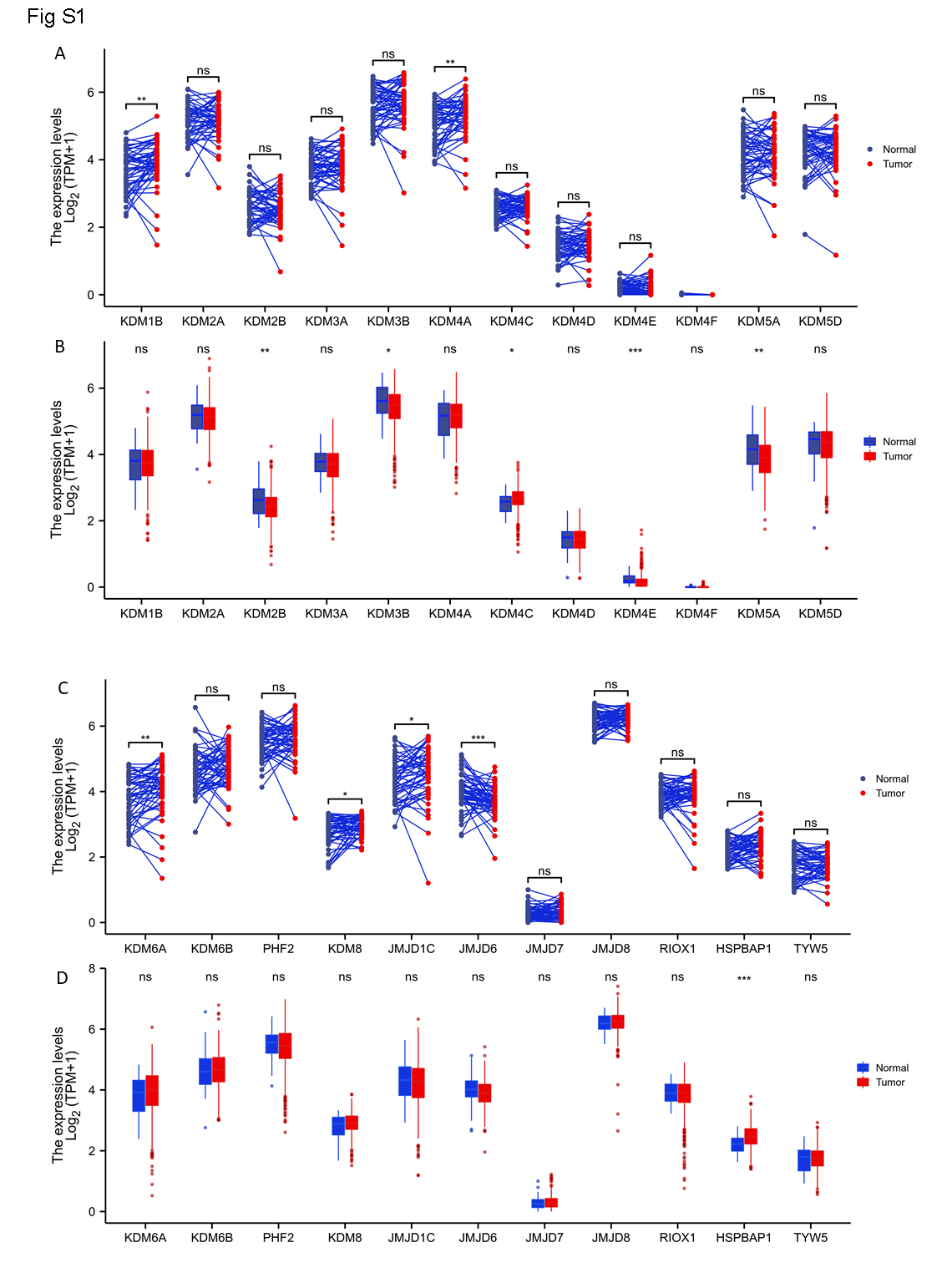

Supplement: Supplementary Figure 1 — RIOX2 expression data in normal and malignant prostate tissues were extracted from the TCGA-PRAD dataset for two types of comparison, case-matched pairwise (panels A & C) and group cohort (panels B & D). Wilcoxon rank sum test, *p < 0.05; **p < 0.01; ***p < 0.001; ns, no significance. [file Image_1.tif]

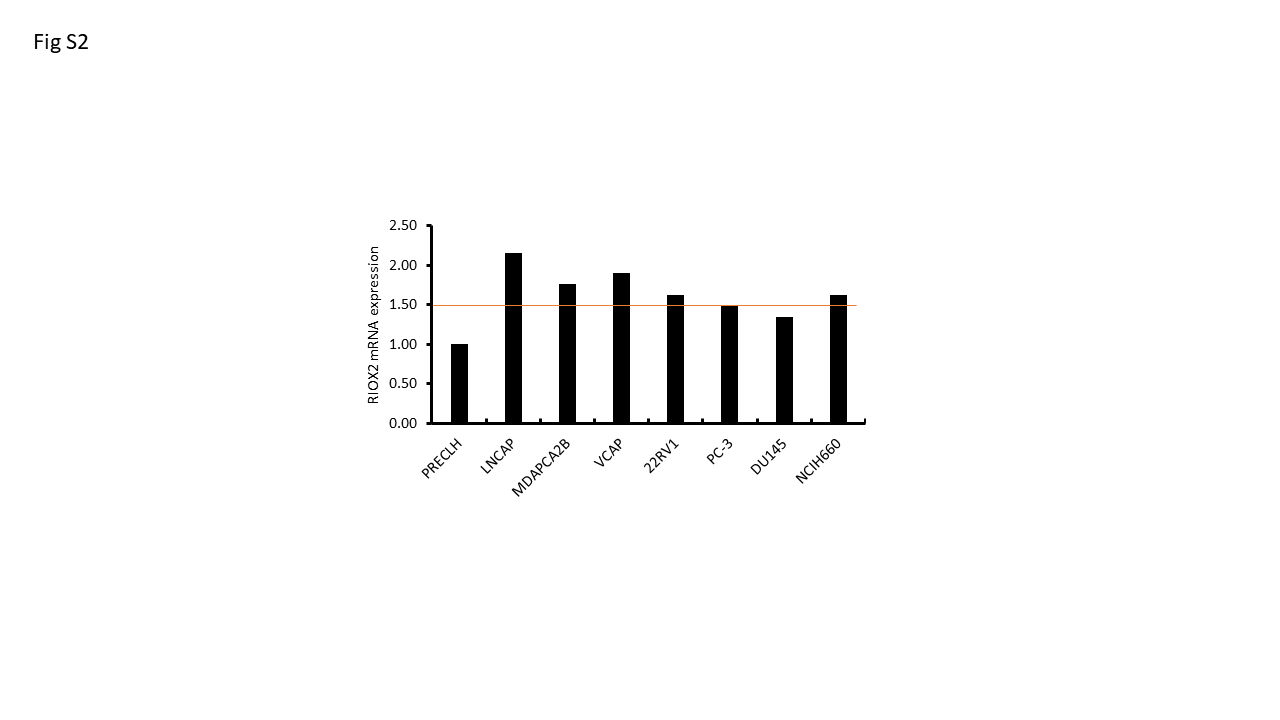

Supplement: Supplementary Figure 2 — RIOX2 expression in normal and malignant prostate cell lines were extracted from the Cancer Cell Line Encyclopedia datasets (51, 52) downloaded from the cBioportal platform. Relative RIOX2 mRNA expression compared to the benign PRECLH cell line was shown after normalized with ACTB expression level. [file Image_2.tif]

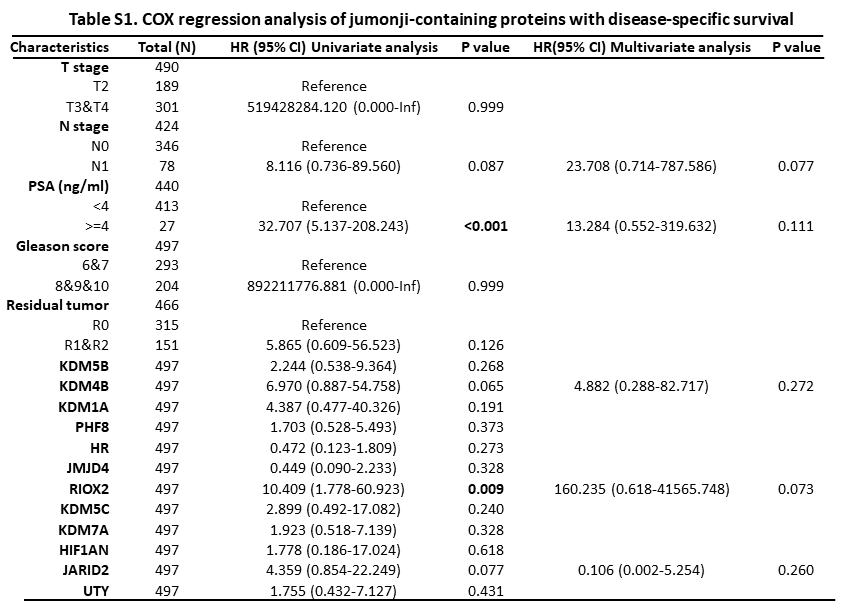

Supplement: Supplementary Table 1 — COX aggression analysis of twelve Jumanji domain-containing protein genes with disease-specific survival outcomes. [file Table_1.docx]
